# Increased fiber density of the fornix in patients with chronic tinnitus revealed by diffusion-weighted MRI

**DOI:** 10.3389/fnins.2023.1293133

**Published:** 2023-12-21

**Authors:** Stephanie Rosemann, Josef P. Rauschecker

**Affiliations:** Laboratory of Integrative Neuroscience and Cognition, Department of Neuroscience, Georgetown University Medical Center, Washington, DC, United States

**Keywords:** tinnitus, diffusion-weighted magnetic resonance imaging, white matter morphology, fiber density, fornix

## Abstract

Up to 45% of the elderly population suffer from chronic tinnitus - the phantom perception of sound that is often perceived as ringing, whistling, or hissing “in the ear” without external stimulation. Previous research investigated white matter changes in tinnitus patients using diffusion-weighted magnetic resonance imaging (DWI) to assess measures such as fractional anisotropy (a measure of microstructural integrity of fiber tracts) or mean diffusivity (a measure for general water diffusion). However, findings overlap only minimally and are sometimes even contradictory. We here present the first study encompassing higher diffusion data that allow to focus on changes in tissue microstructure, such as number of axons (fiber density) and macroscopic alterations, including axon diameter, and a combination of both. In order to deal with the crossing-fibers problem, we applied a fixel-based analysis using a constrained spherical deconvolution signal modeling approach. We investigated differences between tinnitus patients and control participants as well as how cognitive abilities and tinnitus distress are related to changes in white matter morphology in chronic tinnitus. For that aim, 20 tinnitus patients and 20 control participants, matched in age, sex and whether they had hearing loss or not, underwent DWI, audiometric and cognitive assessments, and filled in questionnaires targeting anxiety and depression. Our results showed increased fiber density in the fornix in tinnitus patients compared to control participants. The observed changes might, reflect compensatory structural alterations related to the processing of negative emotions or maladaptive changes related to the reinforced learning of the chronic tinnitus sensation. Due to the low sample size, the study should be seen as a pilot study that motivates further research to investigate underlying white matter morphology alterations in tinnitus.

## 1 Introduction

Tinnitus is the phantom perception of sound, often perceived as whistling, buzzing or hissing “in the ear” without external stimulation. Approximately 10–15% of the adult population and up to 45% of the elderly population suffer from chronic tinnitus (Chang et al., 2019; Jafari et al., 2019; Knipper et al., 2020; Chen et al., 2021; Hu et al., 2021). The perception of the phantom sound can have a debilitating impact on the patients’ quality of life and frequently comes with mental health issues, including increased anxiety and depression, as well as problems in concentration and sleeping (Noreña, 2011; Schecklmann et al., 2014). The permanent tinnitus perception can also lead to tinnitus-related distress (and vice versa) which can be predicted by psychological and socio-demographic measures, such as problems with sleep and concentration or psychological stress (Brueggemann et al., 2022). Additionally, even in tinnitus patients with mild tinnitus-related distress, the presence of psychiatric comorbidities is higher than in healthy controls (Ivansic et al., 2019). It has also been shown that tinnitus is related to a decrease in cognitive abilities, specifically those of executive control of attention (Heeren et al., 2014; Trevis et al., 2016), inhibitory control (Araneda et al., 2015; Trevis et al., 2016; Araneda et al., 2018), and general cognitive abilities like short-term memory, concentration and orientation (Wang Y. et al., 2018). Furthermore, speech comprehension, particularly under adverse listening conditions, such as competing noise, is impaired in tinnitus patients, even if they have normal hearing abilities (Ivansic et al., 2017; Tai and Husain, 2019; Liu et al., 2020). There is currently no effective treatment or cure for tinnitus, and in order to advance treatment options, understanding its pathophysiology is of utmost importance.

Previous research has provided evidence of neural alterations covering both structure and function in tinnitus patients compared to control participants. Changes in white matter morphology are usually investigated using diffusion-weighted magnetic resonance imaging (DWI) and a diffusion tensor imaging (DTI) model to compute measures such as fractional anisotropy (FA, a measure of microstructural integrity of fiber tracts) or mean diffusivity (MD, a measure for general water diffusion) (Khan et al., 2021). Alterations in FA and MD were found in tinnitus patients in regions near the auditory cortex (Crippa et al., 2010; Aldhafeeri et al., 2012; Seydell-Greenwald et al., 2014) and in the inferior colliculi (Crippa et al., 2010; Seydell-Greenwald et al., 2014; Gunbey et al., 2017), as well as in prefrontal cortex (Aldhafeeri et al., 2012), thalamus and limbic areas (Crippa et al., 2010; Aldhafeeri et al., 2012; Gunbey et al., 2017). Other studies reported white matter changes in regions such as the anterior thalamic radiation, the inferior fronto-occipital fasciculus, both the superior and inferior longitudinal fasciculus (Benson et al., 2014; Khan et al., 2021), as well as the anterior corona radiata, the anterior corpus callosum and sagittal strata (Aldhafeeri et al., 2012; Yoo et al., 2016; Khan et al., 2021). Additionally, FA values in amygdala, hippocampus, parahippocampus and prefrontal cortex correlated negatively with tinnitus-related distress (Gunbey et al., 2017).

Hence, previous research has already shown that changes in white matter morphology can be detected by DWI. We here want to try a different approach by using a fixel-based analysis approach that enables to resolve the multiple fibers problem. One voxel usually contains several white matter tracts each denoted as a fiber population. Thus, there are multiple – even crossing – fibers within a voxel. Standard models (such as DTI models) are not able to capture multiple fibers within a single voxel and hence FA values, for instance, are not fiber-specific. A fixel-based analysis, by contrast, enables us to analyze specific fiber pathways even within regions that contain crossing fibers. The main aim of a fixel-based analysis is to investigate whether changes in intra-axonal volume are related to decreased fiber density (FD, changes in tissue microstructure), to reduced fiber bundle cross section (FC, macroscopic changes), or to a combination of both (FDC). FD is sometimes also referred to as axonal density (Assaf et al., 2008) or neurite density (Zhang et al., 2012) and relates to the intra-axonal volume. FC describes the axon diameter or the number of voxels that the fiber bundle occupies (Raffelt et al., 2017). A reduction in FC may indicate that the fiber bundle is atrophic. FDC is a combination of FD and FC indicating a change in intra-axonal volume as well as axon diameter. Reduced fiber density along with accumulated axon loss, such as in Alzheimer’s disease, might be reflected in a decrease in FDC (Raffelt et al., 2017).

The purpose of this project was to investigate changes in white matter morphology (FD, FC and FDC) in tinnitus patients compared to control participants. We further explored whether and how tinnitus distress and cognitive abilities are related to changes in white matter morphology. Our hypotheses were decreased FD, FC and FDC in tracts connecting auditory cortex, thalamic, limbic, and prefrontal brain regions (Crippa et al., 2010; Aldhafeeri et al., 2012; Benson et al., 2014; Seydell-Greenwald et al., 2014; Yoo et al., 2016; Gunbey et al., 2017; Khan et al., 2021). Of specific interest are changes in white matter pathways between the auditory cortex, nucleus accumbens, and ventromedial prefrontal cortex, as these components are thought to play a causal role in a prominent frontostriatal gating model of tinnitus (Rauschecker et al., 2010, 2015; Leaver et al., 2011). Further, we expected that decreased cognitive abilities might be related to decreased white matter morphology (FD, FC and FCD), specifically in prefrontal regions (Aldhafeeri et al., 2012; Benson et al., 2014; Rosemann and Rauschecker, 2022, 2023). In addition, we hypothesized that tinnitus distress is negatively correlated with FD, FC and FDC in tracts connecting amygdala, hippocampus, parahippocampus, and prefrontal cortex (Gunbey et al., 2017).

## 2 Methods

### 2.1 Participants

We recruited 20 tinnitus patients and 20 control participants matched in terms of age and sex to participate in the study. The mean age in tinnitus patients was 58.5 (±9.82) years and the mean age in the control group was 57.7 (±10.7). Each group comprised 7 female and 13 male participants. Tinnitus patients and control participants were matched in terms of whether they had hearing loss or not.

Participants were recruited through social networks, advertisements and from our previous studies. Pediatric populations, individuals with HIV, individuals with history of seizures or other neurological disorders, with magnetic resonance imaging (MRI)-incompatible implants, with significant ear asymmetries, those with exposure to loud noise 24 h prior to testing, and pregnant women were excluded. The study was approved by the Institutional Review Board at Georgetown University and conducted in accordance with the Code of Ethics of the World Medical Association (Declaration of Helsinki). All participants gave written and informed consent and were paid for participating in this study.

The study was preregistered on OSF on July 20, 2021 and can be found at https://osf.io/scph4.

### 2.2 Audiometric assessment

**Figure 1:**
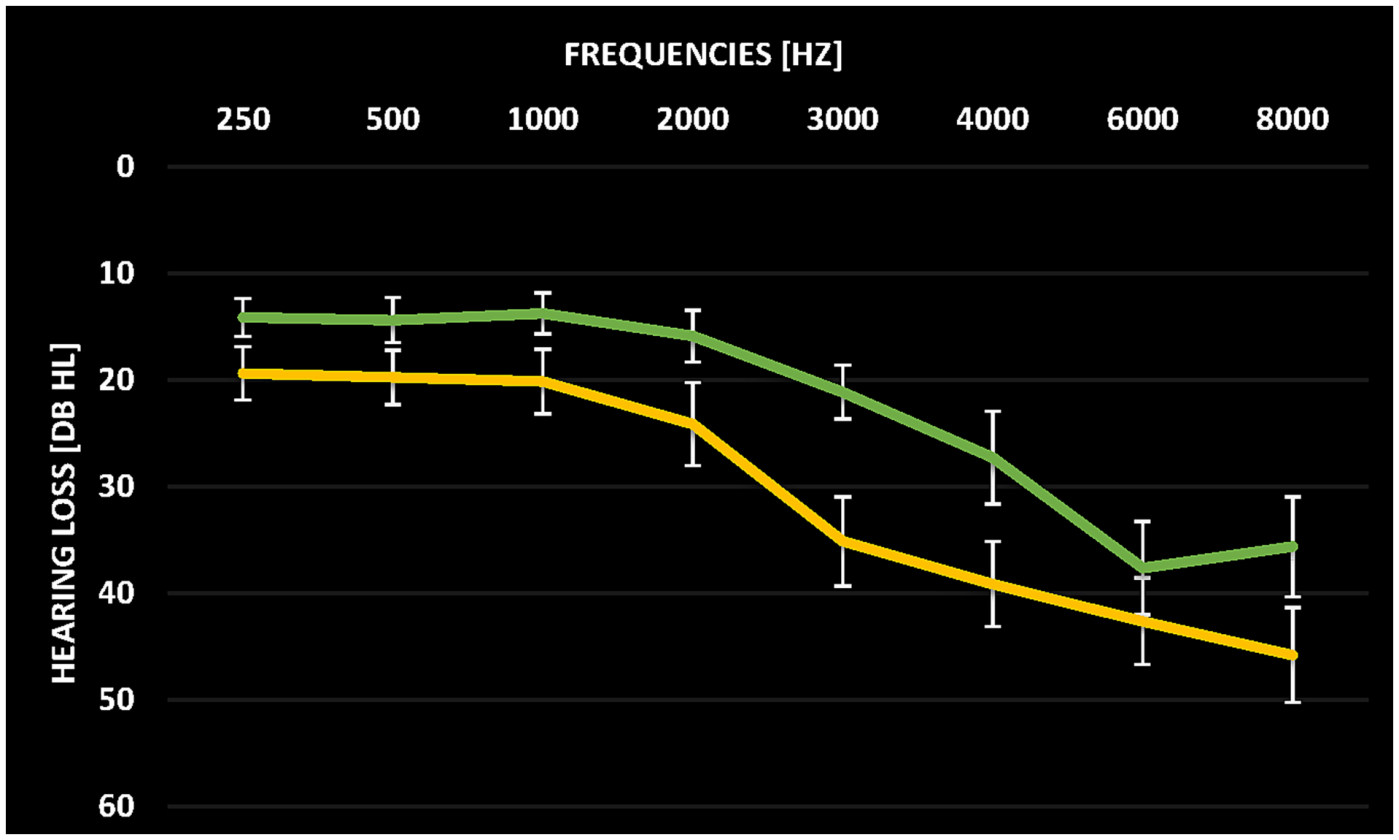
Average pure tone audiograms for tinnitus patients (yellow) and control participants (green) averaged over both ears. Error bars denote standard error of the mean.

The audiometric assessment was conducted at the Division of Audiology and Hearing Research at Medstar Georgetown University Medical Center. Pure-tone thresholds of the frequencies ranging from 250 Hz to 16 kHz were assessed in a sound-proof chamber. Pure-tone audiograms averaged over both ears for the two groups are depicted in Figure 1. Tinnitus patients and control participants were matched in terms of whether they had hearing loss or not. However, the assessed hearing thresholds at 3 kHz and 4 kHz as well as the mean hearing loss between 250 Hz and 8 kHz significantly differed between tinnitus patients and control subjects (*T*(38) = 2.194, *p* = 0.034). Hearing thresholds as quantified by the mean hearing threshold over the frequencies 250 Hz to 8 kHz were, therefore, included in the between-group analysis (see Data analysis).

Further, tinnitus patients underwent an additional assessment to match the frequency and perceived intensity of their tinnitus. The mean perceived pitch frequency of the tinnitus was 8 kHz (range 3–12.5 kHz, *n* = 8 perceived the pitch at 8 kHz and *n* = 7 at 10 kHz) and the mean perceived tinnitus intensity was 5 dB SL. Four tinnitus patients reported a unilateral tinnitus sensation, all others reported a bilateral tinnitus sensation. Tinnitus duration ranged from 6 months to 50 years (mean duration 15.3 ± 12.4 years).

### 2.3 Behavioral assessment

Prior to the MRI, general cognitive abilities were assessed with the Montreal Cognitive Assessment (MoCa) (Nasreddine et al., 2005). The MoCa is a test for early detection of mild cognitive impairment and assesses the subdomains visuospatial functions, naming, attention, language, abstraction, delayed recall and orientation. Working memory was tested with a self-developed two-back task. In this visual task, participants were asked to indicate whether the current number was the same as two numbers before (two-alternative forced-choice task). The duration was 10 minutes (five blocks times 2 min; 20 s break in between the blocks). Stimuli consisted of digits between 1 and 9, and 160 stimuli were presented in total (number of targets was 45). The duration of one trial was 2,700 ms, and the presentation of the stimuli was jittered (jitter was 100 or 300 ms). Presentation of the stimuli was pseudo-randomized in the way that the same amount of targets was present in each block. The order of the blocks was the same for each participant. Stimulus presentation was controlled by Presentation^®^ software (version 22.0; Neurobehavioral Systems, Inc., Berkeley, CA).^1^ The total MoCa score and the accuracy (hits + correct rejections) in the two-back task were used as measures for cognitive abilities and working memory performance in the statistical analysis.

Additionally, participants completed questionnaires assessing anxiety, depression and emotional distress: The Patient Health Questionnaire 9 (Kroenke et al., 2001), the Generalized Anxiety Disorder (Spitzer et al., 2006), and the Hospital Anxiety and Depression Scale (Zigmond and Snaith, 1983). All participants also conducted the Modified Khalfa Hyperacusis Questionnaire (Khalfa et al., 2002). Tinnitus patients further filled in the Tinnitus Handicap Inventory (THI; Newman et al., 1996), the Tinnitus Sample Case History Questionnaire (Langguth et al., 2007) and the Tinnitus Functional Index (TFI; Henry et al., 2016).

### 2.4 Data acquisition

MRI data were acquired by a 3 T whole-body Siemens Magnetom Prisma MRI machine with a 64-channel head coil. Structural images were acquired with a 3-D T1-weighted sequence (MP-RAGE, TR = 1900 ms, TE = 2.52 ms, voxel size = 1.0 × 1.0 × 1.0 mm, flip angle 9 degrees, 160 sagittal slices). Duration was 5 min. DWI data were acquired with a multi-directional diffusion weighting (MDDW) sequence with 6 non-diffusion images, 27 diffusion images with a *b*-factor of *b* = 1,000 s/mm^2^ and 53 diffusion images with a *b*-factor of *b* = 3,000 s/mm^2^ (TR = 4,700 ms, TE = 81 ms, voxel size = 2.0 × 2.0 × 2.0 mm, 70 transversal slices, 53 directions). The duration of the DWI measurement was 8 min. In addition, we acquired resting-state functional MRI data with a duration of 13 min (published in Rosemann and Rauschecker, 2023). The whole duration of the MRI scans was approximately 30 min.

### 2.5 Behavioral data analysis

Statistical analyses for the behavioral data covered between-group comparisons of the cognitive tests and questionnaire scores as well as analysis of correlations between tinnitus characteristics, tinnitus distress and anxiety and depression scores. We computed two-sample *T*-tests or Mann–Whitney-U tests in case of deviation from normal distribution (tested by the Shapiro–Wilk test). This analysis was performed in JASP (JASP Team, 2020; Version 0.14.1). We report standard deviation of the mean, if not indicated otherwise. A threshold of *p* < 0.05 was considered significant.

### 2.6 Fixel-based analysis

The fixel-based analysis to compute FD, FC and FDC metrics was done in MRtrix3 (Raffelt et al., 2017; Tournier et al., 2019).^2^ Preprocessing steps involved denoising, motion and distortion correction, global intensity normalization across all subjects and up-sampling to a voxel size of 1.25 mm^3^. Then, fiber orientation distribution (FOD) estimation based on a group average response function (Raffelt et al., 2012) was computed by the multi-shell multi-tissue (MSMT) constrained spherical deconvolution (CSD) signal modeling approach (Tournier et al., 2013). Next, a study-specific FOD template was computed and all subjects’ FOD images were registered to that template (Raffelt et al., 2011). FOD images were warped to the template space and the fixels were reoriented. Each subject’s fixels were assigned to the template fixels and then FC and FDC metrics were computed (Raffelt et al., 2017). Whole-brain probabilistic fiber-tractography (20 million streamlines) was performed next, followed by applying the spherical-deconvolution informed filtering of tractograms (SIFT) algorithm to reduce the number of streamlines to 2 million. Based on this whole-brain streamlines tractogram, the fixel-fixel connectivity matrix was generated, and then fixel data were smoothed. A General Linear Model was used for the statistical analysis of FD, FC and FDC metrics. We computed between-group comparisons (corrected for hearing loss quantified by the mean hearing loss between 250 Hz and 8 kHz) and multiple linear regression analyses with values from the cognitive tasks (MoCA and two-back task) and tinnitus distress (THI and TFI) within the tinnitus group (corrected for age and sex).

The resulting significant fixels (at a threshold of FWE-corrected *p* < 0.05) were displayed in the mrview tool of the MRtrix3 software. For better visualization, significant fixels were also displayed on a template-derived tractogram (using SIFT to further reduce the number of streamlines to 200.000) that only showed the streamlines of the significant fixels that are color-coded by streamline orientation (Raffelt et al., 2017).

## 3 Results

### 3.1 Behavioral assessments

Mean values (±standard deviation) for tinnitus patients and control participants for all cognitive tests and questionnaires are shown in Table 1. No significant differences between the two groups were obtained for any of the cognitive tasks or the questionnaires (*p* > 0.1). Scores in general cognitive status (MoCA) and working memory (two-back task) were not correlated to the THI, TFI or any values obtained in the tinnitus assessment (*p* > 0.1). Furthermore, mean values for the tinnitus assessment questionnaires were 20 (±11) for the THI and 49.3 (±29.9) for the TFI. Those values were not related to depression and anxiety scores (*p* > 0.1). Similarly, the values in perceived tinnitus pitch and intensity were not related to any of the depression and anxiety scores (*p* > 0.1).

**Table 1:** Overview of cognitive task performance and questionnaire scores assessing anxiety, depression and emotional distress for the tinnitus patients and control participants [mean values ± standard deviation; *T*-values for the between-group comparisons].

	Tinnitus patients (*n* = 20)	Control participants (*n* = 20)	*T*-values	*p*-values
MoCA [score]	27.1 (± 2.14)	26.9 (± 2.37)	0.28	0.781
Two-back task [% accuracy]	89.7 (± 7.6)	86.6 (± 7.1)	1.328	0.192
HADS depression [score]	3.15 (± 2.35)	2.45 (± 2.72)	0.871	0.389
HADS anxiety [score]	5.1 (± 3.21)	3.75 (± 3.68)	1.236	0.224
BDI [score]	6.2 (± 4.58)	4.7 (± 5.74)	0.913	0.367
GAD [score]	1.7 (± 2.11)	1.25 (± 2.0)	0.694	0.492
PHQ [score]	2.0 (± 2.05)	2.1 (± 3.23)	−0.117	0.908
Khalfa hyperacusis [score]	13.05 (± 9.41)	9.1 (± 9.78)	1.302	0.201

No significant differences between the two groups were obtained for any of the cognitive tasks or the questionnaires (all *p*s > 0.1).

### 3.2 DTI data

**Figure 2:**
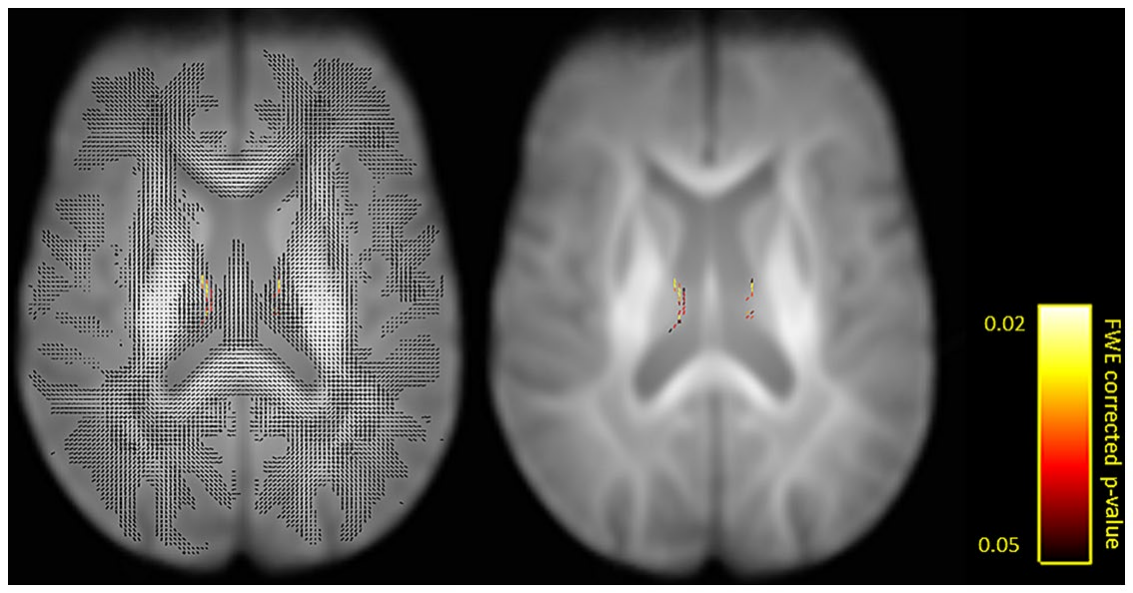
Fixels with a significant increase in fiber density (FD) in tinnitus patients compared to control participants shown on a single axial slice (*z* = 71). Fiber tract-specific inference is enabled by attributing *p*-values to each fixel in crossing-fiber regions. Shown with (left) and for better visibility also without (right) surrounding fixels. Significant fixels are color-coded by p-values (FWE-corrected *p* < 0.05).

**Figure 3:**
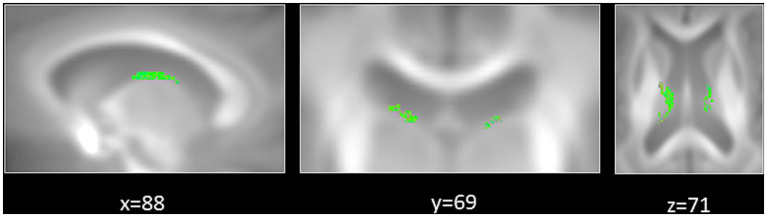
Significant increase in fiber density (FD) in tinnitus patients compared to control participants. Zoomed-in regions show significant fixels that are displayed on a template-derived tractogram (color-coded by streamline orientation; red: left–right; green: anterior–posterior; blue: inferior–superior; FWE-corrected *p* < 0.05).

The first aim of the fixel-based analysis was to compare FD, FC and FDC metrics between tinnitus patients and control participants. For that aim, we computed between-group comparisons controlled for hearing loss. That analysis revealed a significant increase in FD in the fornix in tinnitus patients compared to control participants (Figures 2, 3).

The second aim was to correlate FD, FC and FDC metrics in tinnitus patients with cognitive abilities (MoCA and n-back performance) and tinnitus distress (THI and TFI scores). This analysis showed no significant correlations.

As suggested by two anonymous reviewers, we carried out an exploratory analysis correlating white matter morphology (FC, FD and FDC) with tinnitus duration. For this analysis, we used the duration quantified as months and not years based on the reviewers’ comments. Unfortunately, no significant correlations were obtained in this analysis.

## 4 Discussion

The main aim of this study was to investigate changes in white matter morphology in tinnitus patients compared to control participants. Our hypotheses were decreased FD, FC and FDC in tracts connecting auditory cortex, thalamic, limbic, and prefrontal brain regions (Crippa et al., 2010; Aldhafeeri et al., 2012; Benson et al., 2014; Seydell-Greenwald et al., 2014; Yoo et al., 2016; Gunbey et al., 2017; Khan et al., 2021). Additionally, we explored whether and how tinnitus distress and cognitive abilities are related to changes in white matter morphology. Regarding these relationships, we hypothesized that decreased cognitive abilities would be related to decreased FD, FC and FCD in prefrontal regions (Aldhafeeri et al., 2012; Benson et al., 2014; Rosemann and Rauschecker, 2022, 2023). Further, we hypothesized a negative correlation between tinnitus distress and FD, FC and FDC in tracts connecting amygdala, hippocampus, parahippocampus and prefrontal cortex (Gunbey et al., 2017).

Our results showed a significant increase in FD in the fornix in tinnitus patients compared to control participants. The fornix is the major efferent pathway of the hippocampus, connecting it to the mammillary bodies and the thalamus (Lövblad et al., 2014). It is part of the Papez circuit – an important structure in the limbic system – that is associated with memory and emotion processing (Kamali et al., 2023). Previous research demonstrated tinnitus-related changes in white matter metrics such as decreased fractional anisotropy in limbic regions covering the amygdala and hippocampus (Aldhafeeri et al., 2012; Gunbey et al., 2017) and increased path strength between the auditory cortex and the amygdala (Crippa et al., 2010). Both the amygdala and the hippocampus are thought to be involved in dealing with negative emotions such as depression and anxiety that are frequently reported in tinnitus sufferers (Aldhafeeri et al., 2012). In addition, there is evidence for the involvement of the fornix in several psychiatric disorders such as schizophrenia and bipolar disorders (Koshiyama et al., 2020), depression (Geng et al., 2016; Shim et al., 2023), anxiety (Modi et al., 2013), and post-traumatic stress disorder (Harnett et al., 2020). Interestingly, a reduction of white matter microstructure in the fornix was associated with improved symptoms in post-traumatic stress disorder (Kennis et al., 2015). Other diffusion MRI studies highlighted the role of the fornix in chronic pain (Sean et al., 2023). Hence, the fornix appears to be important in cognitive-affective dysfunctions, and an increased microstructure seems to indicate an increase in symptomatology. We here found an increased fiber density in the fornix in tinnitus patients compared to control participants. This might suggest increased structural connectivity to the hippocampus, possibly involving emotional processing such as awareness, annoyance and even maintenance of the tinnitus sensation. Moreover, the fornix is supposed to be engaged in episodic memory formation (Douet and Chang, 2015) and executive function (Sasson et al., 2013). Damage to the fornix can lead to significant memory and cognitive impairments (Wang J. et al., 2018). On the other hand, plasticity in the microstructure of the fornix has been shown by short-term learning (Hofstetter et al., 2013) and reasoning training (Mackey et al., 2012). Current tinnitus models highlight the mechanism of attentional reinforcement that is related to the sustained perception of the tinnitus signal once it is established (Sedley et al., 2016). Thus, the tinnitus sensation is strengthened by the continuous attention to it and by learning an “expectation” of tinnitus (Sedley et al., 2016). Our results demonstrate an increased fiber density of the fornix in tinnitus patients which might be a sign of – in this case maladaptive – plasticity due to intensified” learning-to-expect” the tinnitus signal. To sum up, we suggest that the strengthened fiber density in the fornix might reflect either a compensatory structural alteration due to the continuous processing of negative emotions – possibly even pointing towards a failure in emotion regulation – related to the tinnitus sensation. Alternatively, the increased fiber density in the fornix might indicate a maladaptive mechanism associated with the reinforced learning of the tinnitus signal that itself leads to the sustained perception of it. Thus, we argue that the observed changes should mostly be seen as *effects* of the tinnitus sensation rather than its *cause*.

Current findings motivate future research investigating white matter morphology in tinnitus that goes beyond the standard DTI model to compute fractional anisotropy or mean diffusivity. Recently, a study showed macrostructural changes in the acoustic radiation in patients with tinnitus using the fixel-based analysis as well (Koops et al., 2021). We here employed a whole-brain and multi-shell multi-tissue CSD signal modeling approach (Tournier et al., 2013) that allowed reliable processing of our higher diffusion data (*b* = 1,000 s/mm^2^ and *b* = 3,000 s/mm^2^). Future studies should invest scan time for the DWI data acquisition to allow the measurement of more than one and especially higher *b*-values (not only *b* = 1,000 s/mm^2^) along with the acquisition of more directions (>30 directions) to reduce bias and increase sensitivity (Glenn et al., 2016; Kincses et al., 2020). Diffusion spectrum imaging as well as diffusional kurtosis imaging might also be suitable to investigate white matter morphology in chronic tinnitus. Extending current knowledge on the neural pathology of tinnitus is of crucial importance and identifying underlying micro- and macrostructural alterations may further aid in advancing treatment options in chronic tinnitus.

## 5 Limitations

It appears that chronic tinnitus is associated with white matter changes that can be observed with a fixel-based analysis. However, we need to point out some limitations of the current study. Contrary to our hypotheses, our fixel-based analysis did not demonstrate any correlation of white matter morphology and cognitive abilities or tinnitus distress in tinnitus patients. One possible reason for the missing relation might be the fact that tinnitus patients did not exhibit cognitive deficits in the MoCA or two-back task. Hence, it is probable that due to normal cognitive functioning, no changes in white matter morphology were obtained. Similarly, our sample did not show significantly higher levels of depression and anxiety compared to the control participants. The reported tinnitus-related distress ranged from slight to catastrophic distress with the mean being in the moderate range (based on TFI and THI scores). Interestingly, the involvement of limbic regions such as the insula and parahippocampus have been shown in mild forms of tinnitus or in patients that successfully habituated to the tinnitus signal and hence reflect lower levels of tinnitus distress (Husain, 2016; Liu et al., 2019). On the contrary, structural MRI studies demonstrated that depression and anxiety scores are major modulators of grey-matter volume in tinnitus patients (Leaver et al., 2012; Besteher et al., 2019). Therefore, it is possible that white matter changes in the limbic system have not been observed due to only moderate degree of tinnitus-related distress or to a lack of comorbid psychiatric disorders in the sample. However, this also highlights the role of psychiatric comorbidities which should be carefully assessed and accounted for in future studies. Another reason for the absent correlations with both cognitive abilities as well as tinnitus distress might be that the sample size for the correlational analysis (*n* = 20) was too small. Thus, this study can only be seen as a pilot study, which might, however, trigger future studies on white matter morphology alterations in tinnitus with larger sample sizes. Moreover, it would also be beneficial to include other cognitive assessments that might be better at targeting possibly affected cognitive processes in tinnitus patients, such as the Attention Network Test (Heeren et al., 2014), a go/no-go task (Araneda et al., 2015) or a Stroop task (Araneda et al., 2018). Moreover, future studies should include speech-in-noise perception in tinnitus patients to assess their possible difficulties in speech comprehension (Ivansic et al., 2017). In addition, it is known that stress and anxiety modulate tinnitus loudness and distress. However, a further limitation of the study is that we did not assess these possible effects, which could start well in advance of the actual study. To further assess stress and distress levels of tinnitus patients, measuring physiological data, such as heart rate variability, blood pressure, or respiratory indicators, might provide additional information. The changes in brain structure we found are mostly thought of as *effects* of the tinnitus rather than a *cause* of it (also considering the extended tinnitus duration). Hence, longitudinal studies are needed in order to provide information on causality of neural alterations in chronic tinnitus.

## 6 Conclusion

The key in advancing treatment options for tinnitus and evaluating the efficacy of tinnitus interventions lies in understanding its underlying pathophysiology. Our study adds evidence of white matter changes in the fornix, indicating higher fiber density in tinnitus patients. We suggest that this might reflect either a compensatory structural alteration from the negative emotional processing of the tinnitus signal or a maladaptive mechanism related to the reinforced learning of the tinnitus sensation. However, future research is needed to unmask the involvement of the fornix in chronic tinnitus and its implications for tinnitus treatment options. Importantly, current results motivate further research employing advanced diffusion imaging techniques to unravel underlying micro- and macrostructural alterations in tinnitus.

## Data availability statement

The datasets presented in this study can be found in online repositories. The names of the repository/repositories and accession number(s) can be found at: OSF project “Tinnitus as a network problem – plasticity in anatomical and functional connectivity”: https://osf.io/2nse8/.

## Ethics statement

The studies involving humans were approved by Institutional Review Board at Georgetown University. The studies were conducted in accordance with the local legislation and institutional requirements. The participants provided their written informed consent to participate in this study.

## Author contributions

SR: Conceptualization, Data curation, Formal analysis, Investigation, Methodology, Visualization, Writing – original draft. JR: Conceptualization, Supervision, Writing – review & editing.

## Footnotes

^1^www.neurobs.com

^2^www.mrtrix.org
